# Supplementary material for: HRAMS Proteomics Insights on the Anti-Filarial Effect of Ocimum sanctum: Implications in Phytochemical-Based Drug-Targeting and Designing
Source: Proteomes. 2024 Dec 27;13(1):2. doi: 10.3390/proteomes13010002 (PMC11755628; doi:10.3390/proteomes13010002)
Supplement: Supplementary file 1 [file proteomes-13-00002-s001.zip › proteomes-3234101-supplementary.pdf]

Supplementary Table S1A. Hydrogen bond statics of Protein models analyzed by VADAR

Target Protein	Mean H-bond Distance		Mean H-bond Energy		Residue with H-Bond	
	Observed	Expected	Observed	Expected	Observed	Expected
ADK	2.2 sd=0.4	2.2 sd=0.4	-1.7 sd=1.0	-2.0 sd=0.8	180 (86%)	155 (75%)
Enolase	2.3 sd=0.4	2.2 sd=0.4	-1.6 sd=1.1	-2.0 sd=0.8	341 (78%)	327 (75%)
GAPDH	2.2 sd=0.4	2.2 sd=0.4	-1.9 sd=1.1	-2.0 sd=0.8	266 (78%)	253 (75%)
HSP70	2.2 sd=0.4	2.2 sd=0.4	-1.7 sd=1.0	-2.0 sd=0.8	522 (80%)	483 (75%)
PGK	2.3 sd=0.4	2.2 sd=0.4	-1.6 sd=1.1	-2.0 sd=0.8	338 (81%)	311 (75%)

Supplementary Table S1B. Protein structure elements of Protein model depicted by VADAR server v1.8

Target Protein	Helix	Beta	Coil	Turn
ADK	124 (59%)	31 (14%)	52 (25%)	12 (5%)
Enolase	187 (42 %)	92 (21%)	157 (36%)	56 (8%)
GAPDH	92 (27%)	125 (36%)	121 (35%)	92 (27%)
HSP70	264 (40%)	184 (28%)	197 (30%)	56 (8%)
PGK	180 (43%)	98 (23%)	137 (33%)	56 (13%)

Supplementary Table S2. Ramachandran plot analysis of targeted protein models by Procheck server

**Target Protein**	**Core region**	**Allowed region**	**Generously allowed region**	**Disallowed region**
**ADK**	96.6 %	2.2 %	0.6%	0.6%
**Enolase**	86.4%	9.2%	2.6%	1.8%
**GAPDH**	93.5%	5.8%	0.3%	0.3%
**HSP70**	90.4%	6.2%	2.1%	1.2%
**PGK**	86.9%	11.2%	1.4%	0.6%

Supplementary Table S3. The docked complex of GAPDH, Enolase, ADK, HSP70, and PGK with different bioactive compounds of OS showing interacting receptor residue, and ligand and protein complex involved in H-bonding.

**Receptor**	***Ocimum sanctum* bioactive compounds**	**Interacting receptor residue**	**Ligand and Protein atoms involved in H-bonding**
**GAPDH**	Eugenol	ASN^{9}, GLY^{10}, PHE^{11}, ASN^{34}, ASP^{35}, PRO^{36}, PHE^{37}, SER^{83}, LYS^{84}, PRO^{86}, ILE^{89}, THR^{103}, VAL^{105}, PHE^{106}	NA
	Kaempferol	ASN^{9}, GLY^{10}, PHE^{11}, GLY^{12}, ARG^{13}, ARG^{16}, ASN^{34}, ASP^{35}, PRO^{36},	ASN^{34}:HD22 – LIGAND:O5<br>LIGAND:H30 – ASN^{9}:OD1

		PHE^{37}, ILE^{38}, MET^{46}, SER^{83}, LYS^{84}, PRO^{86}, ILE^{89}, THR^{103}, VAL^{105}, PHE^{106}	
	Luteolin	ASN^{9}, GLY^{10}, PHE^{11}, ASN^{34} ASP^{35}, PRO^{36}, PHE^{37}, ILE^{38}, MET^{46} SER^{83}, LYS^{84}, ILE^{89}, THR^{103}, VAL^{105}, PHE^{106}	LIGAND:H30 – ASN^{34}:O
	Rosmarinic acid	ASN^{9}, GLY^{10}, PHE^{11}, GLY^{12}, ASN^{34}, ASP^{35}, PRO^{36}, PHE^{37}, ILE^{38}, SER^{83}, LYS^{84}, PRO^{86}, ILE^{89}, SER^{102}, THR^{103}, VAL^{105}, PHE^{106}	LIGAND:H30 – SER^{83}:O<br>LIGAND:H42 – ASN^{35}:OD2
	Rutin	GLY^{12}, ARG^{13}, ILE^{14}, GLY^{15}, SER^{102}, THR^{103}, GLY^{104}, SER^{126}, ALA^{127}, PRO^{128}, SER^{156}, CYS^{157}, THR^{158}, THR^{182}, HIS^{184}, THR^{187}, ALA^{188}, THR^{189}, THR^{216}, ALA^{218}, ALA^{237}, ARG^{239}, ASN^{321}, GLU^{322}, TYR^{325,}	ILE^{14}:H – LIGAND:O16<br>THR^{216}:HG1 – LIGAND:O10<br>ARG^{239}:HH12 – LIGAND:O3<br>ARG^{239}:HH22 – LIGAND:O3<br>ASN^{321}:HD22 – LIGAND:O4<br>LIGAND:H64 – THR^{158}:OG1<br>LIGAND:H72 – SER^{102}:O
	Ursolic acid	GLY^{12}, ARG^{13}, ILE^{14}, GLY^{15}, SER^{102}, THR^{103}, GLY^{104}, SER^{126}, ALA^{127}, PRO^{128}, SER^{156}, CYS^{157}, THR^{158}, HIS^{184}, THR^{187}, ALA^{188}, THR^{189}, SER^{215}, THR^{216}, GLY^{217}, ARG^{239}	SER^{126}:HG – LIGAND:O2
	Albendazole	PRO^{28}, GLY^{29}, SER^{30}, GLY^{31}, LYS^{32}, GLY^{33}, THR^{34}, SER^{49}, GLY^{51}, ARG^{55}, ASP^{105}, TYR^{107}, ARG 140 HIS 143 ARG^{144}, THR^{147}, SER^{148}	**NA**
	DEC	PRO^{28}, SER^{30}, GLY^{31}, LYS^{32}, GLY^{33}, HIS^{47}, SER^{49}, GLY^{51}, ASP^{52}, ARG^{55}, ASP^{105}, TYR^{107}, ARG^{140}, ARG^{144}	NA
**Enolase**	Eugenol	HIS^{44}, LEU^{116}, LYS^{120}, ALA^{123}, LEU^{130}, THR^{379}, GLU^{380}, ASP^{381}, THR^{382}, PHE^{383}, ASP^{386}, ARG^{415}	NA
	Kaempferol	LEU^{116}, LYS^{120}, ALA^{123}, VAL^{124}, GLY^{127}, MET^{128}, PRO^{129}, LEU^{130}, THR^{382}, PHE^{383}, ASP^{386}, ARG^{415}, ILE^{416}, GLU^{419}	ARG^{415}:HH12 – LIGAND:O2<br>ARG^{415}:HH12 – LIGAND:O4<br>ARG^{415}:HH22 – LIGAND:O4<br>LIGAND:H30 – GLY^{127}:O

	Luteolin	LEU^{116}, LYS^{120}, ALA^{123}, VAL^{124}, GLY^{127}, ME^{128}, PRO^{129}, LEU^{130}, THR^{382}, PHE^{383}, ASP^{386}, ARG^{415}, ILE^{416}, GLU^{419}	ARG^{415}:HH12 – LIGAND:O4<br>ARG^{415}:HH22 – LIGAND:O4
	Rosmarinic acid	THR^{41}, LYS^{105}, ASN^{154}, GLY^{156}, SER^{157}, ASP^{209}, GLU^{210}, ASP^{245}, ALA^{247}, ALA^{248}, SER^{249}, GLU^{250}, ASP^{297}, ASP^{322}, LEU^{323}, LYS^{346}, GLN^{349}, ARG^{375}	LYS^{346}:HZ – LIGAND:O2<br>ARG^{375}:HH22 – LIGAND:O7<br>LIGAND:H39 – GLY^{127}:OD1
	Rutin	SER^{37}, GLY^{38}, ALA^{39}, THR^{41}, GLU^{45}, ALA^{46}, LEU^{47}, LYS^{105}, ASN^{109}, ASN^{154}, GLY^{156}, SER^{157}, LEU^{163}, GLU^{167}, ASP^{209}, GLU^{210}, ASP^{245}, ALA^{247}, SER^{249}, GLU^{250}, ASP^{297}, ASP^{322}, LEU^{323}, LYS^{346}, GLN^{349}, ARG^{375}	LYS^{105}:HZ2 – LIGAND:O12<br>LYS^{346}:HZ2 – LIGAND:O9<br>GLN^{349}:H21 – LIGAND:O13<br>LIGAND:H63 – GLU^{210}:OE2<br>LIGAND:H64 – GLU^{167}:OE1<br>LIGAND:H70 – GLU^{349}:OE1<br>LIGAND:H73 – GLU^{250}:OE1
	Ursolic acid	LEU^{116}, LYS^{120}, ALA^{123}, VAL^{124}, GLY^{127}, MET^{128}, PRO^{129}, LEU^{130}, THR^{382}, PHE^{383}, ASP^{386}, ARG^{415}, ILE^{416}, GLU^{419}	LYS^{120}:HZ1 – LIGAND:O2
	Albendazole	ALA^{33}, ALA^{34}, HIS^{44}, LEU^{116}, LYS^{120}, ALA^{123}, PRO^{129}, LEU^{130}, THR^{379}, THR^{382}, PHE^{383}, ASP^{386}, ARG^{415}, ILE^{416}, GLU^{419}	NA
	DEC	THR^{41}, ASN^{154}, GLY^{156}, SER^{157}, GLU^{167}, GLU^{210}, ASP^{245}, ALA^{247}, SER^{249}, GLU^{250}, ASP^{297}, ASP^{321}, ASP^{322}, LEU^{323}, LYS^{346}, ARG^{375}	NA
**ADK**	Eugenol	GLY^{27}, PRO^{28}, PHE^{129}, ASP^{131}, VAL^{132}, SER^{133}, LEU^{137}, LEU^{162}, TYR^{165}, ILE^{166}, THR^{169}, ALA^{170}, VAL^{173}, LYS^{184}	NA
	Kaempferol	PRO^{28}, SER^{30}, GLY^{31}, LYS^{32}, GLY^{33}, THR^{34}, SER^{49}, GLY^{51}, ASP^{105}, TYR^{107}, ARG^{140}, ARG^{144}, THR^{147}, SER^{148}	GLY^{31}:H – LIGAND:O4<br>GLY^{33}:H – LIGAND:O2<br>ARG^{140}:H – LIGAND:O3

	Luteolin	PRO^{28}, GLY^{29}, SER^{30}, GLY^{31}, LYS^{32}, GLY^{33}, HIS^{47}, SER^{49}, ASP^{52}, ARG^{55}, ASP^{105}, ARG^{140}, CYS^{141}, HIS^{143}, ARG^{144}	LYS^{32}:HZ3 – LIGAND:O3<br>GLY^{33}:H – LIGAND:O3<br>ARG^{140}:HE – LIGAND:O6<br>LIGAND:H31 – ARG^{140}:O
	Rosmarinic acid	PRO^{28}, GLY^{29}, SER^{30}, GLY^{31}, LYS^{32}, GLY^{33}, THR^{34}, SER^{49}, GLY^{51}, ASP^{52}, ARG^{55}, TYR^{107}, ARG^{140}, HIS^{143}, ARG^{144}, THR^{147}, SER^{148}, ARG^{150,}	GLY^{31}:H – LIGAND:O1<br>THR^{34}:H – LIGAND:O6<br>ARG^{55}:HH22 – LIGAND:O7<br>SER^{148}:HG – LIGAND:O3<br>LIGAND:H41 – ARG^{49}:OG
	Rutin	PRO^{28}, GLY^{29}, SER^{30}, GLY^{31} LYS^{32}, GLY^{33}, THR^{34}, ASP^{37}, HIS^{47}, SER^{49}, GLY^{51}, ASP^{52}, ARG^{55}, ASP^{105}, TYR^{107}, ARG^{140}, HIS^{143}, ARG^{144}, SER^{148}, GLY^{149}, ARG^{150}, ARG^{161}	LYS^{32}:HZ1 – LIGAND:O8<br>ARG^{55}:HH12 – LIGAND:O10<br>ARG^{55}:HH22 – LIGAND:O10<br>TYR^{107}:HH – LIGAND:O8
	Ursolic acid	PRO ^{28}, SER^{30}, GLY^{31}, LYS^{32}, GLY^{33}, THR^{34}, ASP^{37}, HIS^{47}, SER^{49}, GLY^{51}, ASP^{52}, ARG^{55}, ASP^{105}, TYR^{107}, ARG^{140}, HIS^{143}, ARG^{144}, THR^{147}, SER^{148}	**NA**
	Albendazole	PRO^{28}, GLY^{29}, SER^{30}, GLY^{31}, LYS^{32}, GLY^{33}, THR^{34}, SER^{49}, GLY^{51}, ARG^{55}, ASP^{105}, TYR^{107}, ARG 140 HIS 143 ARG^{144}, THR^{147}, SER^{148}	**NA**
	DEC	PRO^{28}, SER^{30}, GLY^{31}, LYS^{32}, GLY^{33}, HIS^{47}, SER^{49}, GLY^{51}, ASP^{52}, ARG^{55}, ASP^{105}, TYR^{107}, ARG^{140}, ARG^{144}	LYS^{32}:H23 – LIGAND:O81<br>GLY^{33}:H – LIGAND:O1<br>ARG^{144}:HH21 – LIGAND:N2
**PKG**	Eugenol	ALA^{287}, ASN^{289} PHE^{290}, ARG^{291}, GLU^{292}, ALA^{294}, PHE^{296}, LEU^{312}, VAL^{340}, GLU^{342}, TRP^{343,}	NA
	Kaempferol	ASP^{23}, PHE^{24}, ASN^{25}, VAL^{26}, PRO^{27}, HIS^{62}, LEU^{63}, GLY^{64}, ARG^{65}, GLY^{165}, HIS^{168}, ARG^{169}, LYS^{214}, GLY^{371}, GLY^{372}, ASP^{373}, THR^{392}, GLY^{393}, GLY^{394}, GLY^{395} ALA^{396,}	LYS^{214}:HZ3 – LIGAND:O5<br>GLY^{372}:HG1 – LIGAND:O3<br>LIGAND:H31 – LEU^{63}:O

	Luteolin	ASP^{23}, ASN^{25}, PRO^{27}, ARG^{65}, ARG^{121}, GLY^{165}, GLY^{212}, ALA^{213}, LYS^{214}, LYS^{218}, ASN^{335}, GLY^{336}, PRO^{337}, GLY^{371}, GLY^{372}, ASP^{373}, GLY^{393}, GLY^{394}, GLY^{395}, LEU^{398}	ARG^{65}:HH11 – LIGAND:O2<br>ARG^{65}:HH11 – LIGAND:O3<br>LYS^{218}:HZ1 – LIGAND:O5<br>LYS^{218}:HZ1 – LIGAND:O6
	Rosmarinic acid	ASP^{23}, PHE^{24}, ASN^{25}, VAL^{26}, PRO^{27}, LEU^{28} LEU^{63}, GLY^{64}, ARG^{65}, PHE^{74}, GLY^{165}, GLY^{212}, ALA^{213}, LYS^{214}, LYS^{218}, PRO^{337}, GLY^{371}, GLY^{372}, ASP^{373}, THR^{392}, GLY^{393}, GLY^{394}, GLY^{395}, LEU^{398}	ASN^{25}:HD21 – LIGAND:O1<br>ASN^{25}:HD21 – LIGAND:O2<br>ARG^{65}:HH11 – LIGAND:O6<br>ALA^{213}:H – LIGAND:O4<br>LYS^{214}:H – LIGAND:O4<br>LYS^{218}:HZ1 – LIGAND:O3<br>LYS^{218}:HZ1 – LIGAND:O4<br>LYS^{218}:HZ2 – LIGAND:O3<br>GLY^{372}:H – LIGAND:O2<br>LIGAND:H41 – PHE^{24}:O<br>LIGAND:H42 – VAL^{26}:O
	Rutin	ASP^{23}, ASN^{25}, VAL^{26}, PRO^{27}, LEU^{28}, LYS^{29}, HIS^{62}, LEU^{63}, GLY^{64}, ARG^{65}, PHE^{74}, ARG^{121}, GLY^{165}, LYS^{214}, LYS^{218}, GLY^{336}, PRO^{337}, GLY^{371}, GLY^{372}, ASP^{373}, THR^{392}, GLY^{393}, GLY^{394}, GLY^{395}	ASN^{25}:HD21 – LIGAND:O9<br>LEU^{28}:H – LIGAND:O14<br>GLY^{372}:H – LIGAND:O10
	Ursolic acid	ASP^{23}, ASN^{25}, VAL^{26}, PRO^{27}, LEU^{28}, LEU^{63}, GLY^{64}, ARG^{65}, PHE^{74}, ARG^{121}, GLY^{212}, ALA^{213}, LYS^{214}, LYS^{218}, GLY^{336}, PRO^{337}, PHE^{341}, GLU^{342}, GLY^{371}, GLY^{372}, ASP^{373}, THR^{374}, THR^{392}, GLY^{393}, GLY^{394}, GLY^{395}, LEU^{398}	ARG^{65}:HE – LIGAND:O2<br>LYS^{218}:HZ1 – LIGAND:O1
	Albendazole	ASP^{23}, ASN^{25}, VAL^{26}, PRO^{27}, LEU^{63}, GLY^{64}, ARG^{65}, ARG^{121}, GLY^{165}, GLY^{212}, ALA^{213}, LYS^{214}, LYS^{218}, ASN^{335}, GLY^{336}, PRO^{337}, GLY^{371}, GLY^{372}, ASP^{373}, GLY^{393}, GLY^{394}, GLY^{395}	GLY^{395}:H – LIGAND:O3
	DEC	ASP^{23}, ASN^{25}, ARG^{65}, GLY^{165}, GLY^{212}, ALA^{213}, LYS^{214}, LYS^{218}, ASN^{335}, GLY^{336}, PRO^{337}, GLY^{371}, GLY^{372}, ASP^{373}, THR^{392},	LYS^{214}:HZ3 – LIGAND:O1<br>GLY^{395}:H – LIGAND:O1

		GLY^{393}, GLY^{394}, GLY^{395}	
**HSP70**	Eugenol	VAL^{25}, GLU^{26}, ILE^{27}, ALA^{182}, ASP^{186}, ILE^{363}, GLU^{367}, ALA^{368}, TYR^{371}	LIGAND:H22 – VAL^{25}:O
	Kaempferol	ASN^{34}, ARG^{35}, THR^{36}, ARG^{272}, SER^{275}, SER^{276}, THR^{341}, ARG^{342}, GLN^{347}, LYS^{361}, SER^{362}, ILE^{363}, ASN^{364}, PRO^{365}	ARG^{272}:HH22 – LIGAND:O5<br>ARG^{342}:HE – LIGAND:O1<br>ASN^{364}:HD21 – LIGAND:O1<br>LIGAND:H31 – LYS^{361}:O
	Luteolin	ASN^{34}, ARG^{35}, THR^{36}, ARG^{272}, SER^{275}, SER^{276}, THR^{341}, ARG^{342}, GLN^{347}, ILE^{363}, ASN^{364}, PRO^{365}	LIGAND:H30 –SER^{375}:O
	Rosmarinic acid	ASN^{34}, ARG^{35}, THR^{36}, ARG^{272}, THR^{273}, SER^{275}, SER^{276}, THR^{341}, ARG^{342}, GLN^{347}, LYS^{361}, SER^{362}, ILE^{363}, ASN^{364}, PRO^{365}	SER^{376}:HG – LIGAND:O2<br>LIGAND:H38 –SER^{276}:O
	Rutin	ARG^{35}, THR^{36}, ARG^{272}, SER^{275}, SER^{276}, ARG^{299}, LEU^{336}, THR^{341}, ARG^{342}, PRO^{344}, GLN^{347}, LYS^{361}, ILE^{363}, ASN^{364}, PRO^{365}	ARG^{342}:HE – LIGAND:O3<br>GLN^{347}:HE21 – LIGAND:O11<br>ASN^{364}:HD21 – LIGAND:O3
	Ursolic acid	ARG^{35}, THR^{36}, ARG^{272}, SER^{275}, SER^{276}, THR^{341}, ARG^{342}, GLN^{347}, LYS^{361}, ASN^{364}, PRO^{365}	ASN^{364}:HD21 – LIGAND:O2
	Albendazole	TYR^{611}, GLY^{620}, MET^{621}, MET^{625}, PRO^{626}, SER^{627}, PRO^{630}, SER^{634}, THR^{635}, GLY^{638}, ILE^{641}, VAL^{644}	NA
	DEC	TYR^{611}, GLY^{619}, GLY^{620}, MET^{621}, MET^{625}, PRO^{626}, SER^{627}, ALA^{629}, PRO^{630}, SER^{634}, THR^{635}, GLY^{638}, ILE^{641}, VAL^{644}	NA

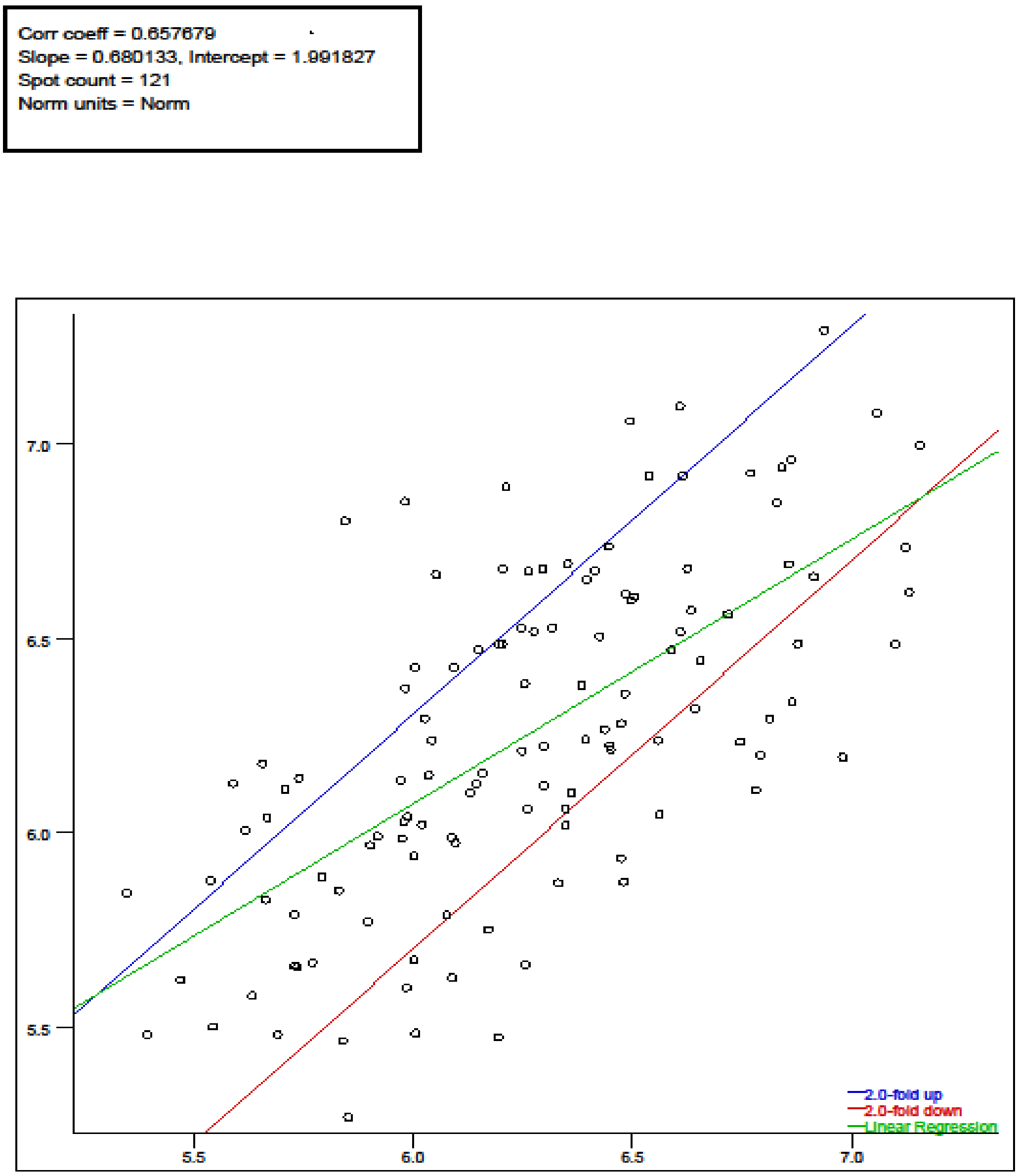

Supplementary figure S1. Correlation graph of spot matching. Analysis of protein spots in adult female *S. cervi* parasites after EOS treatment. Light blue region: upregulated spots and red region: downregulated spots.

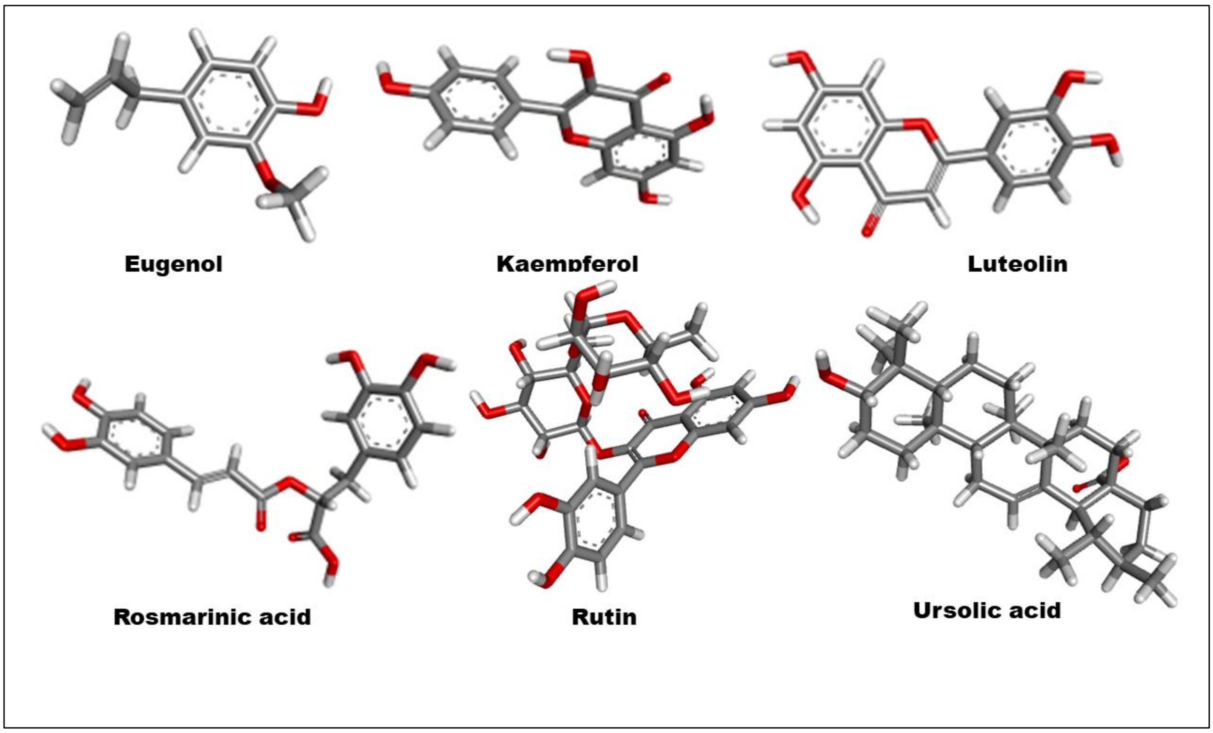

Supplementary figure S2. *Ocimum sanctum* bioactive compounds structure used in molecular docking study retrieved from PubChem database.
